# Screening of Three Echinoderm Species as New Opportunity for Drug Discovery: Their Bioactivities and Antimicrobial Properties

**DOI:** 10.1155/2018/7891748

**Published:** 2018-03-01

**Authors:** Loredana Stabili, Maria Immacolata Acquaviva, Rosa Anna Cavallo, Carmela Gerardi, Marcella Narracci, Patrizia Pagliara

**Affiliations:** ^1^Dipartimento di Scienze e Tecnologie Biologiche ed Ambientali, Università del Salento, Via Prov.le Lecce-Monteroni, Lecce, Italy; ^2^Istituto per l'Ambiente Marino Costiero, U.O.S. di Taranto, CNR, Via Roma 3, Taranto, Italy; ^3^Istituto di Scienze delle Produzioni Alimentari, U.O.S. di Lecce, Via Prov.le Lecce-Monteroni, Lecce, Italy

## Abstract

Echinoderms are a renewable resource with an economic value due to their increasing demand as food and/or source of bioactive molecules exerting antitumor, antiviral, anticoagulant, antioxidant, and antimicrobial activities. In this framework, the present study is aimed at investigating the antibacterial, antioxidant, and hemolytic activities in the three Echinoderm species* Echinaster sepositus*,* Arbacia lixula*, and* Sphaerechinus granularis*. The sea star* E. sepositus* showed lysozyme-like activity (mean diameter of lysis of 13.4 ± 0.2 mm), an antimicrobial activity against the human emerging pathogens* Staphylococcus aureus*,* Pseudomonas aeruginosa*, and* Candida famata*, and a strong lytic activity (100 ± 0.05%) towards the human red blood cells. Furthermore* A. lixula* and* E. sepositus* had the highest antioxidant activity (1792.75 ± 233.7 and 1765.65 ± 484.58 nmolTE/mL, resp.). From toxicological assays, it was shown that* E. sepositus* was not toxic towards HeLa cells and* Vibrio fischeri*, encouraging the exploitation of this species in the pharmaceutical field. Therefore, our findings have implications due to the ongoing explosion of antibiotic-resistant infections because of the new opportunistic pathogens and the need to discover antibacterial agents with new modes of action. Also the recorded antioxidant activity taking into account the need to find natural antioxidants useful for human health is intriguing.

## 1. Introduction

Echinodermata is a phylum containing approximately 7,000 living species and is a remarkable economic renewable resource: holothurian and echinoid cultures indeed are an important economic activity especially in Asia. With the increasing demand for sea urchin roe and trepang (a generic name for sea cucumbers), commercial culture venues have grown in order to maintain the demands for these organisms [1–3]. Moreover, recently echinoderms have received great attention as an unexploited source of new bioactive molecules with important antimicrobial, antiviral, antiprotozoal, antifungal, and antihelminthic anticancer activities suggesting their potential applicability for drug discovery. The peculiarities of these molecules are stability, activity at low temperature, and specificity of action. Often these molecules are part of their innate immune system. As invertebrates lacking adaptive immunity, echinoderms are an excellent model for studying innate immunity. Their defense mechanisms are mediated by cellular and humoral responses [4]. Cellular responses are carried out by several types of coelomocytes, which are free roaming cells circulating in the coelomic cavity and able also to infiltrate tissues and organs [4]. Different morphologically distinguishable cell types can be found in the coelomic fluid: phagocytic amebocytes, colored and colorless spherula cells, vibratile cells, hemocytes, and crystal cells [5, 6]. Their action includes phagocytosis of cellular debris, the formation of a layer of cells at the site of injury, cell clumping, and the formation of capsules around ingested parasite [7]. Humoral immunity is mediated by a broad variety of secreted molecules that can be found in the coelomic fluid. These molecules are capable of recognizing foreign matter, neutralizing, or destroying pathogens, inducing or enhancing cellular responses (opsonization), and helping during wound healing [4, 5, 8]. These secreted immune molecules include lectins, hemolysins, cytokines, the complement protein family, and antimicrobials that have been the subjects of extensive research, even to the point of potential medical applications [9]. As an example, sea cucumber has been valued in Chinese medicine for hundreds of years as a cure for a wide variety of ailments [9]. More recently, compounds exerting antimicrobial, antiviral, and antitumor activity, mainly from sea cucumbers and starfish, have been isolated, leading to a growing interest for the discovery of immunostimulatory activities useful for human health [10, 11]. In particular, much attention has been addressed to antimicrobial peptides (AMPs); small proteins of the innate immune response were evolutionary conserved [12]. AMPs act on Gram-negative and Gram-positive bacteria and envelop viruses, fungi, and even transformed or cancerous cells. In echinoderms, also lysozymes and fragments of larger proteins display antimicrobial activities [13]. Lysozyme is a ubiquitous enzyme widely distributed in the animal kingdom and induces cell lysis of the bacterial cell wall by the hydrolysis of *β*1–4 glycoside bond between NAM and NAG. There are several lysozymes or lysozyme-like proteins identified from coelomic fluid, coelomocytes, and other tissues of echinoderms [13].

The search for bioactive compounds with antioxidant activity is also intriguing considering the positive association between nutraceutical or functional food and human health. In particular, natural antioxidant compounds play an important role as health-protecting factors from free radicals and ROS (reactive oxygen species) effects [14]. Recently, an interest in natural antioxidants has increased because they are widely distributed and safer than synthetic antioxidants. The antioxidants may play a major therapeutic role in human disease causing also the regression of premalignant lesions and inhibiting their development into cancer [15].

Although most efforts have been devoted to terrestrial plants and microorganisms, little attention has yet been paid to marine invertebrates as potential sources of antioxidants [16]. In this framework with the aim of discovering new bioactive molecules, we focused on three echinoderm species investigating their antibacterial antioxidant and hemolytic activities. In particular, the antibacterial activity against some new emerging human pathogens was investigated. The search of novel molecules as alternatives to conventional antibiotics is needed taking into account the ongoing explosion of antibiotic-resistant infections due to new opportunistic pathogens that continue to plague global health care.

## 2. Materials and Methods

### 2.1. Echinoderm Collection

Adult specimens of the sea star* Echinaster sepositus* and of the sea urchins* Sphaerechinus granularis* and* Arbacia lixula* were collected, by using SCUBA equipment, in a coastal area of the Northern Ionian Sea (Porto Cesareo, Lecce, Italy) at a depth of 5–10 m. After the sampling, the animals were kept in circulating seawater, transported to the laboratory, and utilized for the sequent assays.

### 2.2. Samples Preparation

The coelomic fluid (CF) of* E. sepositus* was obtained by a transverse cut of the sea star arms. From the two sea urchins, coelomic fluid was harvested by bleeding through the peristomial membrane. For each species, the CF samples from 20 individuals were collected and then divided into two aliquots. The first aliquot was employed for the evaluation of cell vitality and light microscope observations. The second aliquot was immediately centrifuged at 400 ×g for 10 min at 4°C. The supernatant (CF) of the different individuals was pooled and employed for the evaluation of the lysozyme-like activity and antibacterial, antioxidant, and hemolytic activities. The pelleted coelomocytes were resuspended in distilled water, pooled, and sonicated on ice with an ultrasound probe (Sonifer sonicator Model 250/240, Brain Ultrasonic Corporation) for 4 min at 50% duty cycles. Sonicated samples were checked under the microscope to ensure cell breakage and centrifuged at 12,000 ×g for 30 min. The supernatants reported as coelomocyte lysates (CL) were used for the biological assays.

### 2.3. Cell Vitality and Microscopic Observations

To evaluate cell vitality, an aliquot of 1 mL of each individual CF from each species was rapidly poured into 1 mL of ice-cold filtered (0.22 *μ*m) seawater (FSW) with 30 mM EDTA. Cell viability has been evaluated using the Trypan blue exclusion method. For this, 10 *μ*L of 0.8% Trypan blue in FSW-EDTA was added to 90 *μ*L of CF, loaded into a hemacytometer, and observed under a light microscope (Nikon Eclipse 50i). Bright cells were counted as live and blue cells were considered dead. At the same time, the presence and the normal morphology of each cell type were monitored.

### 2.4. Lysozyme-Like Activity

To detect lysozyme-like activity, inoculated Petri dishes were used as standard assay [17]. Briefly, 700 *μ*L of 5 mg/mL of dried* Micrococcus lysodeikticus* cell walls (Sigma) was diluted in 7 mL of 0.05 M PB-agarose (1.2%) (pH 5.2) and then spread on a Petri dish. When the agarose gel has solidified, 6.3 mm diameter wells were sunk and filled with 30 *μ*L of sample (CF and CL of each species). The diameter of the cleared zone, due to the lysis of bacterial cell walls, of five replicates for each sample, was recorded after overnight incubation at 37°C. The diameter of the cleared zone was then compared with those of a reference sample represented by hen egg-white lysozyme (Merck) used at a concentration ranging from 0.2 mg/mL to 1.5 mg/mL and producing diameters of lysis comprised between 1.5 and 10.5 mm.

### 2.5. Bacteria and Fungi

To test the antimicrobial activity of the examined species, the following emerging human pathogenic bacteria which are indicator organisms commonly used in programs to monitor antibiotic resistance and kindly furnished by the Microbiology Laboratory of Ospedale “Vito Fazzi,” Lecce (Italy), were employed:* Pseudomonas aeruginosa*,* Enterococcus *sp., and* Staphylococcus aureus*. In addition, the antimicrobial assay was carried out on the clinical isolates of human pathogenic fungi:* Candida albicans* and* Candida famata*. Strains were routinely maintained at +4°C as already described by Stabili et al. [18] and subcultured on a fresh appropriate agar plates 24 h prior to any antimicrobial test.

### 2.6. Disc Diffusion Assay for Antimicrobial Activity

To measure the antimicrobial activity of the examined species on the selected microbial strains the same methodology described by Stabili et al. [18] was used. Briefly, sterile paper discs were impregnated with test samples (CF or CL of each species at the same proteic concentration of 0.2 mg/mL) while discs impregnated with an equivalent volume of FSW were used as negative controls. For each assay, agar plates were seeded, using a sterile swab to give a uniform covering, with 100 *μ*L of test bacteria or fungi suspension adjusted to 10^8^ CFU (colony forming unit)/mL, and impregnated discs and controls were laid onto the surface (indirect cell counting method with absorbance value at 540 nm; Perkin Elmer model Lambda 3B spectrophotometer). After incubation for 24 hours at 37°C, the clear zone around each disc due to microbial growth inhibition was observed and the diameter of the clear zone was measured. The diameter of microbial growth inhibition was then calculated as total diameter of clear zone.

### 2.7. Hemolytic Activity

Hemolytic activity was assayed as previously described by Pagliara and Canicattì [19]. Briefly, suspensions of human red blood cells (HRBC) (2.5% in Tris buffer: Tris HCl 0.05 M, 0.15 M NaCl pH 8) were used to test the ability of CF and CL samples to exert a lytic activity. The test was performed in triplicate by adding one hundred microliters of each sample (protein concentration 0.2 mg/mL) to an equal volume of HRBC suspension in glass tubes with U bottom. The test tubes were incubated for 60 min at 37°C and then centrifuged for 5 min at 1500 ×g. One milliliter of Tris buffer was added to the supernatant in order to obtain an adequate amount of volume for spectrophotometric evaluation (541 nm) of the hemoglobin content. As controls, 100 *μ*L of HRBC suspension was mixed with an equal volume of Tris buffer. The degree of hemolysis was calculated by the formula: [(absorbance of sample − absorbance of control)/absorbance of total hemolysis] × 100. Total hemolysis (100%) was achieved by adding 100 *μ*L of distilled water to the same volume of the red cell suspension.

### 2.8. Antioxidant Activity

The antioxidant activity was assayed as previously described by Stabili et al. [20] by the Oxygen Radical Absorbance Capacity (ORAC) and the Trolox Equivalent Antioxidant Capacity (TEAC) assays. In particular, for ORAC, the method [21] samples were diluted with 75 mM phosphate buffer (pH 7.4). The assay was carried out in black-walled 96-well plates (Greiner-Bio One) and each well contained a final volume of 200 *μ*L. To each well, 20 *μ*L of sample and 120 *μ*L of fluorescein (FL; 70 nM final concentration) were added and the plate was incubated at 37°C for 15 min. The AAPH (60 *μ*l; 12 mM final concentration) was added to each well and fluorescence intensity was estimated using an Infinite 200 Pro plate reader (Tecan, Männedorf, Switzerland), every minute for a total of 80 min using an excitation wavelength of 485/9 nm and an emission wavelength of 535/20 nm. A standard curve was constructed using 6-hydroxy-2,5,7,8-tetramethylchroman-2-carboxylic acid (Trolox, Sigma-Aldrich, 1.5–10.5 *μ*M). A blank (fluorescein + AAPH) using phosphate buffer instead of the antioxidant solution was carried out in each assay. Results were calculated, using Magellan v 7.2 software (Tecan, Switzerland), on the basis of the difference in area under the curve between the control and the sample and expressed as *μ*moles of Trolox equivalents (TE) per ml of CF or CL. All the reaction mixtures were prepared in triplicate and at least three independent assays were performed for each sample.

The TEAC assay was performed by the classical method [22] modified with minor modifications. Briefly 2,2′-azinobis (3-ethylbenzothiazoline-6-sulfonic acid) diammonium salt (ABTS, Sigma-Aldrich) radical cations were prepared by mixing an aqueous solution of potassium persulfate 2.45 mM (final concentration) and an aqueous solution of ABTS 7 mM (final concentration) and were allowed to stand in the dark at room temperature for 12–16 hours, before use. The ABTS radical cation solution was diluted in PBS (pH 7.4) to an absorbance of 0.40 at 734 nm. Trolox was used as antioxidant standard and a standard calibration curve was constructed for Trolox (0–16 *μ*M). After addition of 200 *μ*L of diluted ABTS to 10 *μ*L of Trolox standard or extracts diluted in PBS, in each well of a 96 well-plate (Costar), the absorbance reading at 734 nm was taken 6 min after initial mixing using an Infinite 200 Pro plate reader (Tecan, Männedorf, Switzerland). Appropriate solvent blank was run in each plate. The CF and CL of each species were assayed at least at three separate dilutions and in triplicate. The percentage inhibition of absorbance at 734 nm is calculated and plotted as a function of concentration of Trolox and the TEAC value expressed as Trolox equivalent (in *μ*molar) per ml of CF and CL, using Magellan v7.2 software.

### 2.9. Protein Content

Protein concentration of samples was determined by the method of Bradford [23], with bovine serum albumin (Bio-Rad) as standard. All the data were normalized at the same protein concentration.

### 2.10. Toxicity Assays

#### 2.10.1. HeLa Cells Culture Conditions and Treatments and MTT Assay

HeLa cells were cultured in sterile conditions in 25 or 75 cm^2^ plastic flasks with D-MEM, supplemented with 10% (v/v) FBS, 2 mM L-glutamine, 100 *μ*g/mL penicillin/streptomycin, and maintained in 5% CO_2_ at 37°C (Thermo Forma Scientific incubator). For propagation, cells were detached and harvested with a 0.3% (v/v) trypsin solution and then transferred to new flasks every 2-3 days (70–90% confluence). The culture medium was changed every 2 days. All experiments were performed between passage 3 and passage 10 propagation. For assays, CF and CL samples were added to the cell culture medium. Control cells were incubated with fresh medium.

The MTT assay was used to evaluate the effects of* E. sepositus* CF and CL on mitochondrial activity and cellular viability. Cells were seeded in 96-well plates (20 × 10^3^ cells/well) and incubated for 24 h at 37°C. After incubation, the medium was removed and replaced with a medium containing CF and CL samples. After treatment, MTT solution (5 mg/mL in sterile filtered PBS, pH 7.4) was added to each well to reach a final concentration of 0.5 mg MTT/mL, and plates were incubated at 37°C for 3 h. The dark-blue formazan crystals were then solubilized by cell lysis with 200 *μ*l/well 2-propanol/HCl 4N, and absorbance was measured at 550 nm with a microplate reader. Data were reported as % of control and were the mean (±SEM) of 8 samples replicates of each treatment. Three independent experiments were performed.

#### 2.10.2. Microtox® Bioassay

The Microtox Basic Test (BT) was performed according to standard operating procedure [24]. The bacteria* (Vibrio fischeri)* were obtained from AZUR Environmental as freeze-lyophilized cells. In the BT, the CF and CL samples of* E. sepositus* were diluted 1 : 10 using the diluent reagent (Microtox™ Diluent). The light emission of the bacteria was measured after 5, 15, and 30 min and compared to an aqueous standard control. The tests were performed at 15°C and pH 8.0 ± 0.5 with control and replicated three times for each sample. All the measurements were performed by using the M500 luminometer. The instrument was interfaced with PC operating with the Microtox Omni 1.16 software for Windows 98 for acquisition and data handling.

### 2.11. Statistical Analysis

All data were analyzed by using the StatSoft STATISTICA v. 6.0 [25] (2001).

## 3. Results

### 3.1. Cell Vitality and Microscopic Observations

The Trypan blue exclusion test revealed a coelomocytes vitality of 91.4 ± 26.1 in* E. sepositus*, 88.3 ± 35.3 in* A. lixula*, and 90.2 ± 23.3 in* S. granularis*.

**Figure 1:**
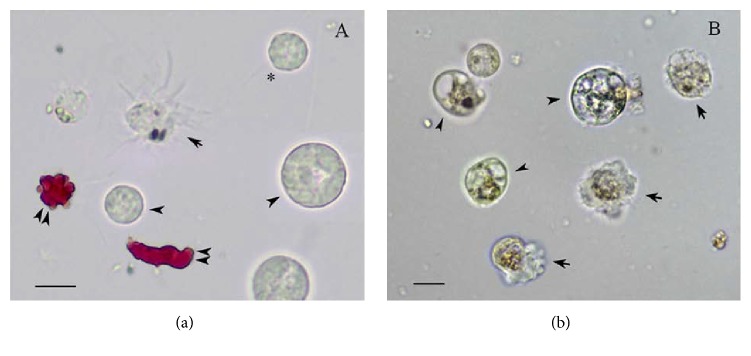
(a) Microscopic images of freshly isolated coelomocytes from* S. granularis* and (b) from* E. sepositus*: arrow, amebocytes; arrowhead, colorless spherula cells; double arrowhead, red spherula cells; asterisk, vibratile cells. Scale bar corresponds to 10 *μ*m.

By observation at light microscopy, we pointed out that, in each sample, diverse cell types with their characteristic heterogeneous size and morphology could be found. In particular, in the two sea urchins,* A. lixula* and* S. granularis* (Figure 1(a)), the coelomic fluid was characterized by the presence of red spherula cells with their peculiar amoeboid movement determining rapid changes in shape. Colorless spherula cells and amebocytes were also present; moreover, in very freshly samples, vibratile cells, rapidly swimming through the fluid by a long flagellum, were observed. In* E. sepositus* (Figure 1(b)), most of the same cell types have been observed with a prevalence of amebocytes and spherula cells. Vibratile cells have also been detected.

### 3.2. Lysozyme Activity

**Figure 2:**
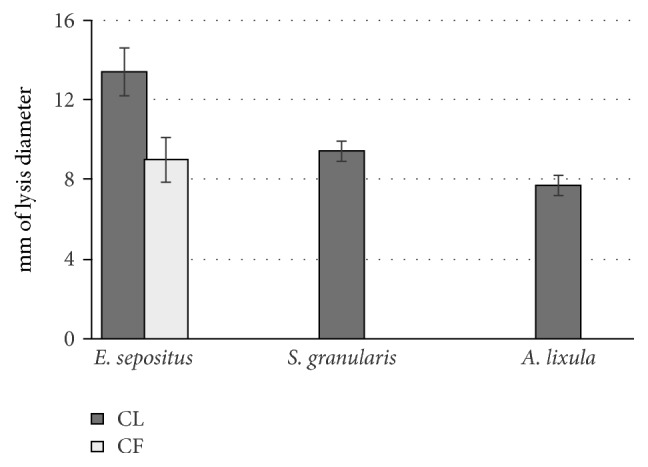
Lysozyme-like activity in coelomocytes lysate (CL) and cell free coelomic fluid (CF) of the three examined echinoderms.

In Figure 2, the lysozyme-like activity of the CL and CF in the examined species is reported. As regards CL, the highest activity was recorded for* E. sepositus* with a mean diameter of lysis of 13.4 ± 0.2 mm corresponding to 1.82 mg/mL of hen egg-white lysozyme, followed by* S. granularis* (diameter of lysis = 9.4 ± 0.5 mm) and* A. lixula* (diameter of lysis of 7.7 ± 0.5 mm). For CF, a valuable activity was recorded only in the case of* E. sepositus* producing a diameter of lysis of 9.0 ± 1.1 mm.

### 3.3. Antimicrobial Activity towards Emerging Pathogens

**Figure 3:**
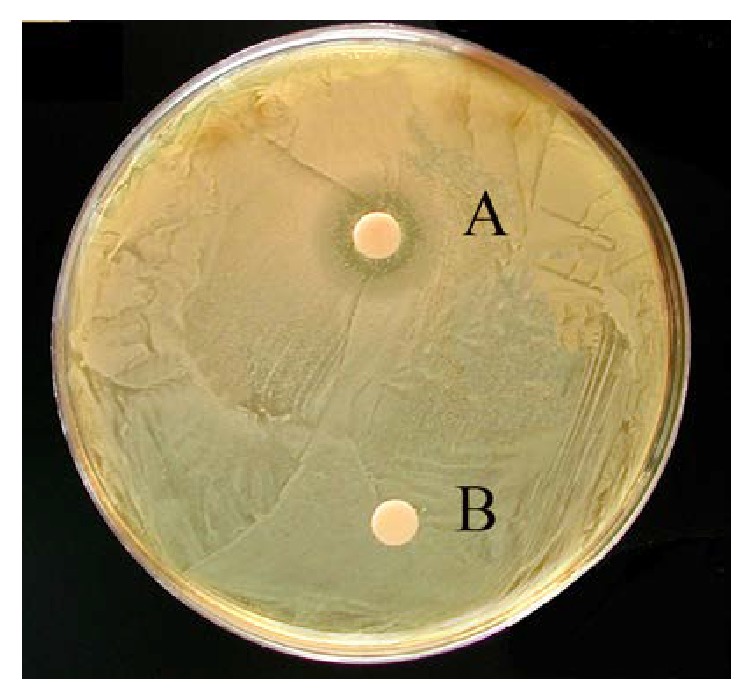
Disc diffusion assay. CL of* E. sepositus* against* Staphylococcus aureus* (A = disc impregnated with CL; B = negative control).

All the three echinoderm species used in this study were screened for antibacterial activity towards some human emerging pathogens. In particular, tests were performed against* Enterococcus *sp.,* P. aeruginosa*, and* S. aureus* and on the clinical isolates of human pathogenic fungi* C. albicans* and* C. famata*. The diameters of inhibition zones were used as a measure of the degree of the antimicrobial activity on each strain. The CL of* E. sepositus* exerted an antimicrobial activity against* S. aureus* (diameter of growth inhibition = 14 ± 0.5 mm) (Figure 3),* P. aeruginosa* (7.5 ± 0.2 mm), and* C. famata* (8 ± 0.3 mm) (Table 1). None of the samples tested demonstrated inhibition against the other bacterial and fungal strains.

### 3.4. Hemolytic Activity

The hemolytic activity of* E. sepositus* CL was very high, inducing 100 ± 0.05% of HRBC hemolysis, while the CF did not possess this activity. An opposite result was obtained for* S. granularis*: in this species, the CF possess a strong lytic activity (86 ± 0.03%), while only 11 ± 0.01% of hemolysis was exerted by CL.* Arbacia lixula* did not show any activity neither in the CF nor in the CL.

### 3.5. Antioxidant Activity

The antioxidant activity of the CL and CF of the three examined species tested by TEAC and ORAC assays is reported in Table 2. Antioxidant capacity measured by TEAC assay was higher than that measured by ORAC assay for almost all the samples. In particular, the highest activity was recorded by TEAC assay for* E. sepositus* and* A. lixula* CL (1965.65 ± 484.58 and 1792.75 ± 233.7 nmolTE/mL sample, resp.). Also the activity observed in the* S. granularis* CL was remarkable. The results obtained by TEAC assay in the CF samples were around 140 nmolTE/mL sample except for* S. granularis* that showed the lowest antioxidant activity by both ORAC and TEAC assay.

### 3.6. Toxicity Assays

Since* E. sepositus* CL showed the highest values for the screened activities in the light of its potential employment as natural source of bioactive compounds, we verified the nontoxicity of the samples by the classical MTT test and the specific Microtox test against* Vibrio fischeri*. MTT test revealed that the sea star cell lysate proved to be nontoxic towards HeLa cells. This result was also corroborated by Microtox data revealing no acute toxicity.

## 4. Discussion

In the present study, we screened three echinoderm species widespread in the Mediterranean area such as* Echinaster sepositus*,* Sphaerechinus granularis*, and* Arbacia lixula* as potential source of antibacterial and antioxidant compounds. From the obtained results, some interesting issues can be inferred.

A strong lysozyme-like activity was evidenced in* E. sepositus*. Lysozymes are enzymes widely distributed throughout the animal kingdom, able to damage the bacterial cell wall catalyzing the hydrolysis of the *β*1–4 glycoside bond between NAM and NAG. In this way, they act as nonspecific innate immunity molecules against the invasion of bacterial pathogens [26]. There are several lysozymes or lysozyme-like proteins identified from coelomic fluid and coelomocytes of echinoderms [13]. The lysozyme-like activity evidenced in* E. sepositus* is noteworthy as potential novel antibacterial compound at the time when multidrug-resistant pathogens are on the rise. This emergence leads to the need for new antibacterial agents with fundamentally different modes of action than those of traditional antibiotics [27, 28]. Bacterial cell wall hydrolases (BCWH) are among the most promising candidates and lysozyme was recently chosen as a model protein [29, 30]; thus, lysozyme from* E. sepositus* could represent a great opportunity in drug systems as a new antimicrobial agent.

It is worth noting that an antibacterial activity of the* E. sepositus* coelomocytes lysate against some new emerging human pathogens was observed for the first time. In particular, the antimicrobial activity was exerted against* S. aureus*,* P. aeruginosa*, and* C. famata*. These microorganisms may become dangerous in particular conditions (body debilitated, etc.) and assume different characteristics of pathogenicity compared to those known. These nosocomial isolates show increasing resistance towards antibiotics representing a great challenge for the management of hospital-acquired infections and the modern pharmacology.* Staphylococcus aureus* is indeed notorious for its ability to become resistant to antibiotics,* P. aeruginosa* develops antimicrobial resistance rapidly, which complicates medical treatment of infections [31], and* C. famata* belongs to the nonalbicans* Candida* (NAC) species. Most of these exhibit primary resistance or reduced susceptibility towards antifungals [32]. Previously, several studies demonstrated that echinoderm coelomocytes lysates and coelomic fluid possess activity against potential pathogenic bacteria, fungi, and even tumor cells. In particular, coelomic fluid from* Echinus esculentus* possesses bactericidal activity against* Pseudomonas *sp. [33]. In the sea urchin* Paracentrotus lividus,* coelomocytes induced growth inhibition against several* Vibrio* sp. including* V. alginolyticus* [34, 35], presented activity against both Gram-positive and Gram-negative bacteria and fungi [36], and showed a cytotoxic activity against rabbit erythrocytes and the K562 tumor cell line [37]. Thus, our findings about antimicrobials from echinoderms may be a valuable therapeutic support opening new perspectives for the utilization of new and still unexploited sources of drugs. Moreover,* E. sepositus* CL exerted an high lytic effect on human red blood cells. This is an interesting observation since hemolytic activity appears to be common in other echinoderm extracts that show high antibacterial activity [38]. On account of these observations the same effector was supposed to be responsible for the two observed activities as a consequence of the transmembrane channels formation [39]. Lytic activity indeed is usually assayed on vertebrate red cells; however, other kinds of cells, including bacteria, are lysed. Thus, also bacteria represent useful targets to demonstrate killing properties of the lytic system as already suggested by Canicattì [39]. In addition, as the lytic activity is known to be also directed against mammalian malignant cells including human leukemia [13], further studies will be also undertaken to explore the potential antitumor activity of the studied echinoderm species.

In addition, in the coelomic fluid and circulating cells of all the examined species, an antioxidant activity was first recorded. The highest values were in the cell lysate of* E. sepositus* and* A. lixula*. In echinoderms, an antioxidant activity was already evidenced in the viscera of the Atlantic sea cucumber* Cucumaria frondosa* and in digestive tract and noncommercial grade gonads of green sea urchin* Strongylocentrotus droebachiensis* [40]. Moreover, in the crude ethanol extract of the starfish* Luidia maculata, *a good antioxidant activity was observed by Suguna et al. [41]. In all these cases, the activity was assayed in tissue extracts and there are few studies only on sea cucumbers demonstrating the coelomic fluid as a good source of antioxidants [42–44]. Due to differences in sample preparation method, data here obtained are not comparable with the antioxidant activity observed in the other investigated echinoderms. Up to now, available studies on aquatic animals have reported data with significant antioxidant ability, only for protein hydrolysates [45]. For this reason the herein highest recorded values (about 1970 nmolTE/mL sample) are comparable only to the well-known natural antioxidant compounds such as the orange juice (1130–3840 nmolTE/mL) and the red wine (500–6000 nmolTE/mL) [46, 47]. It is known from the literature that antioxidants play an important role in the protection of human body against damage by ROS [48]. These molecules indeed induce oxidative damage of DNA and other cellular components leading to cancer, degenerative, and cardiovascular related mutations. Consequently, the intake of natural antioxidant has been associated with reduced risk of cancer and other diseases associated with oxidative damage [49]. In this scenario, the here evidenced activity indicates that the examined echinoderms and in particular* E. sepositus* could represent a new source of natural antioxidants of marine origin.

In conclusion, this study points out the potential of* E. sepositus* cell lysate as promising source of antimicrobial and antioxidant compounds. Obviously, before its exploitation, several toxicity assays will be undertaken. The first performed toxicological tests revealed that* E. sepositus* samples are not toxic towards HeLa cells and* Vibrio fischeri*, encouraging looking at this echinoderm species as a new tool for biotechnological applications in the pharmaceutical and nutraceutical field.

## Figures and Tables

**Table 1:** Antimicrobial activity of CL and CF from the examined echinoderm species.

Microbial strain	Diameter of growth inhibition (mm)					
	*E. sepositus *		*A. lixula*		*S. granularis*	
	CL	CF	CL	CF	CL	CF
*Enterococcus *sp.	0	0	0	0	0	0
*Pseudomonas aeruginosa*	7.5 ± 0.2	0	0	0	0	0
*Staphylococcus aureus*	14 ± 0.5	0	0	0	0	0
*Candida albicans*	0	0	0	0	0	0
*Candida famata*	8 ± 0.3	0	0	0	0	0

**Table 2:** Antioxidant activity of CL and CF of *A. lixula*, *S. granularis*, and *E. sepositus*.

Sample	TEAC	ORAC
	(nmolTE/mL sample)	(nmolTE/mL sample)
CL		
*A. lixula*	1079.725 ± 9.440	1792.75 ± 233.699
*E. sepositus*	832.475 ± 28.956	1965.65 ± 484.58
*S. granularis*	435.757 ± 27.595	671.2 ± 140.290
CF		
*A. lixula*	88.385 ± 1.770	140.500 ± 4.767
*E. sepositus*	85.270 ± 1.440	140.345 ± 3.444
*S. granularis *	64.43 ± 0.978	46.05 ± 1.768

Data are the mean ± SD (*n* = 3); TE = Trolox equivalent.
